# Case report: Reducing the duration of positive-pressure ventilation for large mediastinal masses

**DOI:** 10.3389/fcvm.2022.947847

**Published:** 2022-08-09

**Authors:** Zaili Zhang, Meiru Jiang, Xijia Sun, Wenfei Tan

**Affiliations:** Department of Anesthesiology, The First Affiliated Hospital of China Medical University, Shenyang, China

**Keywords:** large mediastinal mass, positive-pressure ventilation, sternotomy, hyoxemia, case report

## Abstract

Large mediastinal masses (MMs) are rare and present some challenges in hemodynamic and airway management under general anesthesia. Multiple studies have reported cardiopulmonary collapse during general anesthesia. Maintenance of spontaneous ventilation, avoidance of muscle relaxants, and awake-intubation were usually recommended during general anesthesia for high-risk patients with large MMs. However, the recent notion challenged the classic teaching that maintaining spontaneous ventilation is superior to positive-pressure ventilation (PPV). In our case reports, we present two patients with large MMs during general anesthesia. In the first case, a 21-year-old male was administered a muscle relaxant during induction, followed by PPV, but his blood oxygen saturation decreased to 40% after 20 min. Finally, his oxygen saturation was restored by a sternotomy rather than by cardiopulmonary bypass (CPB) by femoral vascular intubation. In the second case, a 33-year-old male was also administered a muscle relaxant during induction followed by PPV, but for him, sternotomy was immediately performed, with stable blood oxygen saturation. Both patients recovered well and were discharged from hospital a week after surgery. Therefore, we present a recommendation that patients with large MMs could undergo PPV after the administration of a muscle relaxant during induction, but the cardiothoracic surgeon should immediately cleave the sternum.

## Introduction

Perioperative management for patients with large mediastinal masses (MMs) is challenging. There has been a focus on the mechanisms of central airway obstruction and cardiovascular instability in recent years. Several studies have recommended maintenance of spontaneous ventilation and awake intubation during general anesthesia for high-risk patients with large MMs (1, 2). Despite more and more guidelines for the management of these patients, perioperative complications still exist. The aim of this article is to provide recommendations, including some experiences and lessons, with regard to the management of patients with large MMs. From our cases, it can be said that reducing the duration of positive-pressure ventilation (PPV) by emergency sternotomy is feasible.

## Case presentation

Two high-risk patients with large MMs undergoing tumorectomy by thoracotomy at the First Affiliated Hospital of China Medical University received general anesthesia. No significant abnormalities had been observed on the pre-surgery laboratory tests (blood routine examination, liver function, renal function, hydrogen ion concentration in blood, coagulation function, and so on) and the electrocardiogram (ECG). Neither patient had a prior underlying medical history.

**FIGURE 1:**
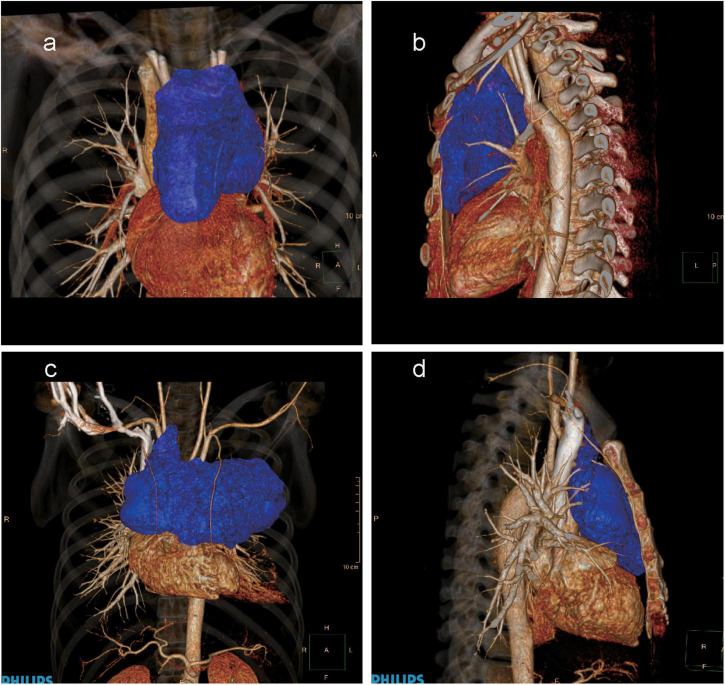
Three-dimensional computed tomography (CT) images of the large anterior mediastinal mass. **(a)** The anteroposterior CT image of the first patient (tumor represented in blue). **(b)** The lateral CT image of the first patient (tumor represented in blue). **(c)** The anteroposterior CT image of the second patient (tumor represented in blue). **(d)** The lateral CT image of the second patient (tumor represented in blue).

The first case was of a 21-year-old male admitted to our hospital because of a large MM (10.19 cm × 5.92 cm, Figures 1a,b); he was a high-risk patient. On 21 April 2020, he was admitted with no obvious symptoms and signs, but with a large MM shown on chest computed tomography (CT) at a local hospital. He had no history of drug allergies, contagious diseases, blood transfusion, surgery, or any other diseases. On the initial visit to the Cardiac Surgery department, his vital signs were as follows: temperature (T), 36.2°C; blood pressure (BP), 115/69 mmHg; heart rate (HR), 90 beats/min; and SPO_2_, 98%. His general condition was not bad, and he had no obvious abnormality on cardiopulmonary auscultation. A diagnosis of a high-risk large anterior MM (tracheobronchial compression >50%) was made. The arch of the aorta was slightly compressed and pushed backwards, and the innominate vein and pulmonary artery were also slightly compressed. On 7 May 2020, the patient was not able to lie down prior to anesthesia without significant dyspnea. His initial vital signs in the operating room were as follows: BP, 130/80 mmHg; HR, 120 beats/min; and SPO_2_, 91%. After the previous chest CT was carefully read, the patient was administered a muscle relaxant during induction followed by PPV. His blood oxygen saturation remained stable after PPV but gradually decreased to 40% after 20 min without significant changes in BP. The cardiothoracic surgeon performed cardiopulmonary bypass (CPB) by femoral vascular intubation, but the blood oxygen saturation only increased to 70%. Finally, the blood oxygen saturation increased to 100% after the cardiothoracic surgeon quickly cleaved the sternum. The pathological diagnosis of the anterior MM after surgery was T lymphoblastic lymphoma. The absence of significant neurological injury after surgery for the patient was due to the prompt rescue of the entire procedure.

The second case was of a 33-year-old male with a large MM (19.1 cm × 9.4 cm, Figures 1c,d); he was a high-risk patient too. On 15 February 2021, he was admitted with dyspnea and chest pain. He had no history of drug allergies, contagious diseases, blood transfusion, surgery, or any other diseases. On the initial visit to the Cardiac Surgery department, his vital signs were as follows: T, 36.6°C; BP, 139/78 mmHg; HR, 92 beats/min; and SPO_2_, 98%. The breath sounds in the right lung were clear on auscultation, and the breath sounds in the left lung were weak, with audible rales. The patient had grade 3/6 systolic jet murmurs in the second intercostal space at the right margin of the sternum. The diagnoses were high-risk large anterior MM (tracheobronchial compression >50%), pericardial effusion, and pleural effusion. The left brachiocephalic vein was compressed and narrowed. The patient’s pericardial effusion was improved preoperatively by pericardiocentesis. His initial vital signs in the operating room were as follows: BP, 120/70 mmHg; HR, 80 beats/min; and SPO_2_, 98%. All puncture and disinfection procedures were performed under local anesthesia. Finally, the patient’s sternum was immediately cleaved by the cardiothoracic surgeon after routine anesthesia induction. The patient was administered a muscle relaxant during induction followed by PPV for short periods, and he had a stable blood oxygen saturation. Ultimately, resection of the large MM was successfully performed following median sternotomy; the pathological diagnosis of the anterior MM after surgery was yolk sac tumor of the mediastinum.

Collectively, both patients recovered well and were discharged from hospital a week after surgery. Both patients reported a comfortable experience after the surgery process. In addition, they presented with no neurological injury after surgery.

## Discussion

Patients with large MMs pose significant challenges due to the possible occurrence of respiratory insufficiency and hemodynamic decompensation. Li et al. (3) described that patients might be distributed into different risk categories, including low risk for asymptomatic patients, intermediate risk for those with tracheobronchial compression <50%, and high risk for those with a tracheobronchial compression >50%. Experts generally agree that low-risk patients tolerate general anesthesia without problems, while intermediate- or high-risk patients need an individualized approach. In the classic teaching, maintaining spontaneous ventilation throughout the procedure and finding a rescue position are the keys to management for intermediate- or high-risk patients (4, 5). Spontaneous ventilation can increase the transpleural pressure gradient, which can distend the intrathoracic airways and prevent collapse (6).

A recent notion, published in the *New England Journal of Medicine*, challenged the classic teaching that maintaining spontaneous ventilation is superior to PPV (7). In addition, a prospective observational study also recommended that, in adult patients with large MMs and tracheobronchial compression, PPV and muscle relaxants could be instituted without compromising central airway patency (8). We also support the notion above. However, some additional supplements should been recommended.

According to the analysis of the first case, blood oxygen saturation remained stable during the initial 20 min of PPV, and emergency sternotomy was an effective method for alleviating hypoxemia. It is suggested that PPV for short periods and emergency sternotomy are feasible for these patients. In addition, hypoxemia may be due to compression of the pulmonary artery by a large MM rather than airway collapse. For several authors, CPB is definitively the recommended modality (9). However, in the first case, hypoxemia was not significantly improved by CPB but by emergency sternotomy.

**FIGURE 2:**
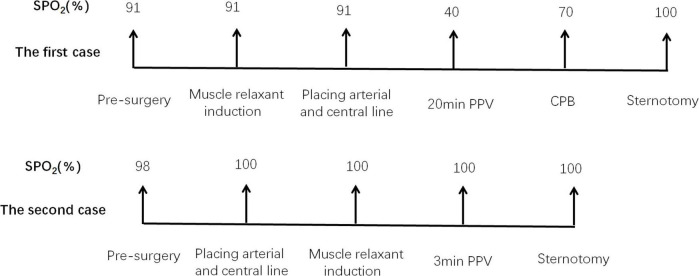
Summary of the entire process. PPV, positive-pressure ventilation; CPB, cardiopulmonary bypass.

The first case had a few deficiencies. A large growing MM could not be accurately evaluated by chest CT one month before surgery. It is regrettable that we did not re-examine the chest CT and assess the symptoms. If all puncture and disinfection procedures were performed under local anesthesia, the patient’s sternum was immediately cleaved by the cardiothoracic surgeon after muscle relaxant administration and PPV for short periods, and the whole anesthesia procedure may have been quite successful. As a result of the anesthesia lessons learned from the first case, we reduced the duration of PPV by performing emergency sternotomy in the second case. There is no doubt that the whole process was successful in the second case (Figure 2).

Based on our cases and the relevant literature, we recommend that patients with large MMs undergo PPV after induction with muscle relaxant administration, but the cardiothoracic surgeon should immediately cleave the sternum. The protocol was as follows: all puncture and disinfection procedures were performed under local anesthesia; the patient was administered a muscle relaxant during induction, and PPV for short periods; and the cardiothoracic surgeon immediately cleaved the patient’s sternum and finished the tumorectomy. The anesthetic induction protocols for this study were consistent with those of Hartigan et al. (7, 8); however, reducing the duration of PPV by emergency sternotomy after administration of a muscle relaxant during induction was also our concern suggestion. Most patients with large MMs will not only have difficult induction of anesthesia but the subsequent maintenance of anesthesia and surgery are challenging too. Hartigan et al. emphasized that the duration of observation for each phase in their study was relatively brief, so they could not confirm the results of prolonged PPV. Therefore, there is no definite time for the PPV before sternotomy, but the less time the better the results.

## Conclusion

In conclusion, we recommend that patients with a large MM should undergo PPV for short periods after the administration of a muscle relaxant during induction, but the cardiothoracic surgeon should cleave the sternum immediately to relieve compression.

## Ethics statement

Written informed consent was obtained from the individual(s) for the publication of any potentially identifiable images or data included in this article.

## Author contributions

ZZ: conceptualization, investigation, and writing – original draft. MJ: project administration and investigation. XS and WT: conceptualization and writing – review and editing. All authors contributed to the article and approved the submitted version.
